# Supplementary material for: Targeting the Achilles’ Heel of Multidrug-Resistant Staphylococcus aureus by the Endocannabinoid Anandamide
Source: Int J Mol Sci. 2022 Jul 14;23(14):7798. doi: 10.3390/ijms23147798 (PMC9319909; doi:10.3390/ijms23147798)
Supplement: Supplementary file 1 [file ijms-23-07798-s001.zip › ijms-1795558-supplementary.pdf]

# Supplementary Figures

# Targeting the Achilles' Heels of Multidrug-Resistant *Staphylococcus aureus* by The Endocannabinoid Anandamide

**Ronit Vogt Sionov, Shreya Banerjee, Sergei Bogomolov, Raphael Mechoulam and Doron Steinberg**

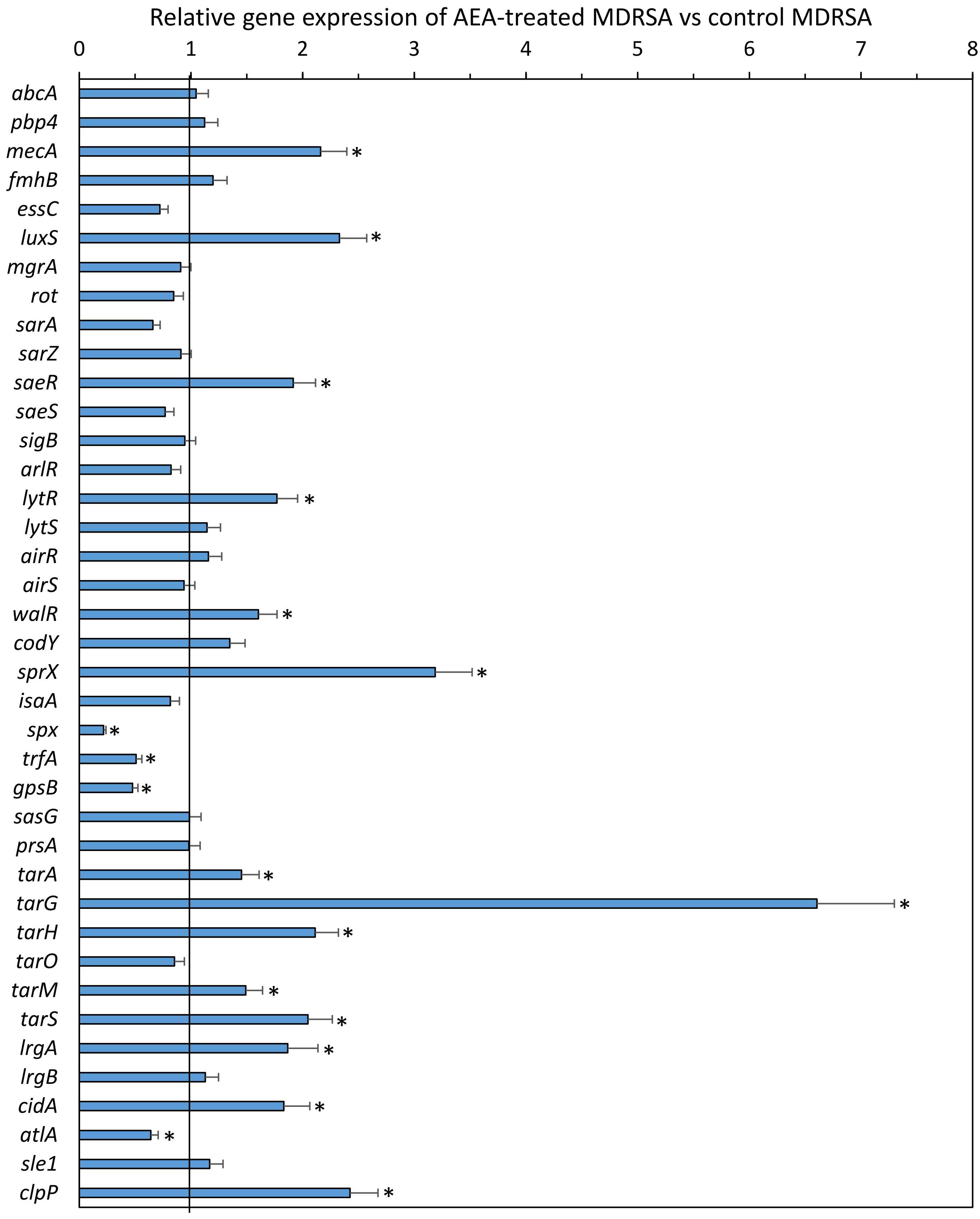


**Supplementary Figure S1. Effect of AEA on gene expression relevant to antibiotic resistance and biofilm formation.** MDRSA CI-M was incubated in the absence or presence of 50 µg/ml of AEA for 2 h, and then the relative gene expression was determined by quantitative RT-PCR. The data presents a representative experiment where two AEA-treated samples were calculated against two control samples, and using the following 9 housekeeping genes: *gmk*, *glyA*, *gyrA*, *gyrB*, *proC*, *recF*, *rho*, *rpoB*, and *asnC*. *$p<0.05$. The relevant function of these genes are described in *Supplementary Table S1*. Other genes are published in Banerjee *et al*. **Sci. Rep.** 11: 8690, 2021.

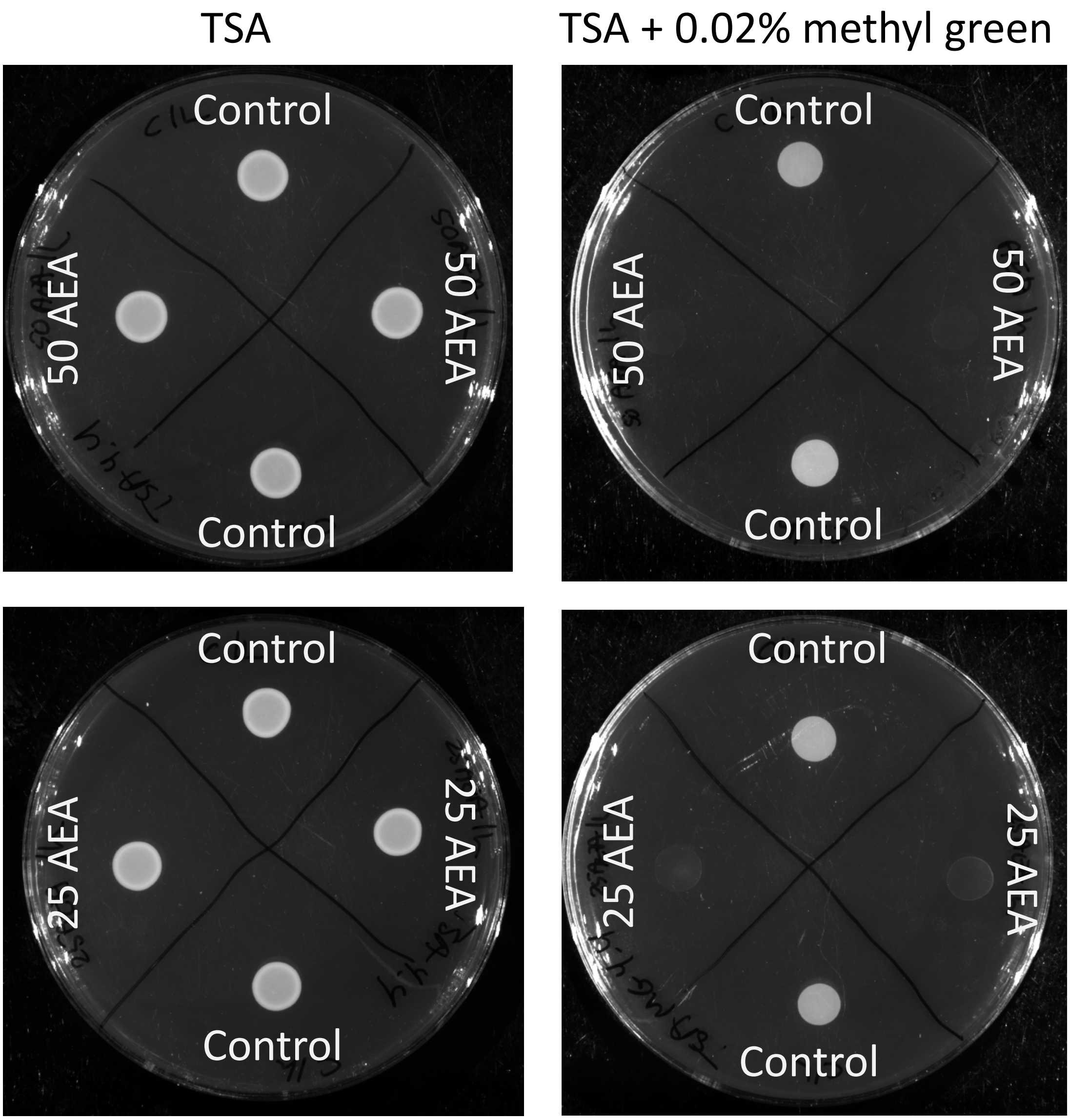


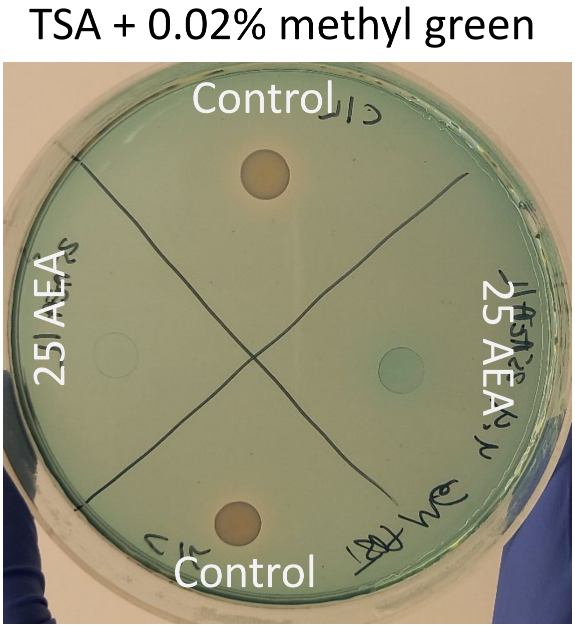


**Supplementary Figure S2. AEA treatment increases the susceptibility to methyl green.** MDRSA CI-M was exposed to 50 or 25 µg/ml AEA for 1 h in TSBG, and then 10 µl was spotted on TSA or TSA with 0.02% methyl green, and the plates were incubated overnight at 37°C.

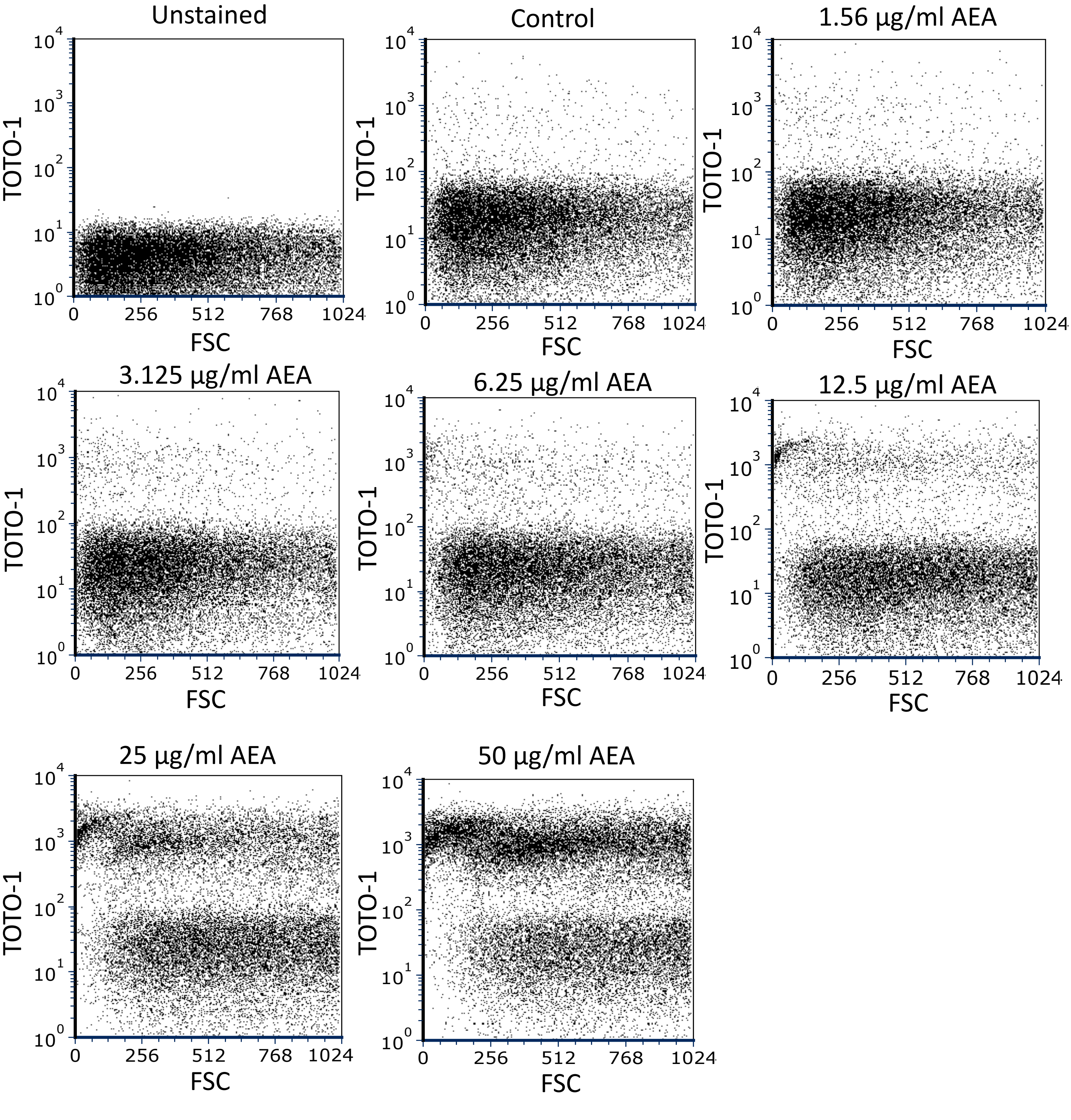


**Supplementary Figure S3. AEA increases cell-bound extracellular DNA.** MDRSA CI-M was exposed to 50 µg/ml AEA for 2 h in TSBG at 37°C, washed in PBS and then exposed to 2 µM TOTO-1 for 20 min at room temperature.

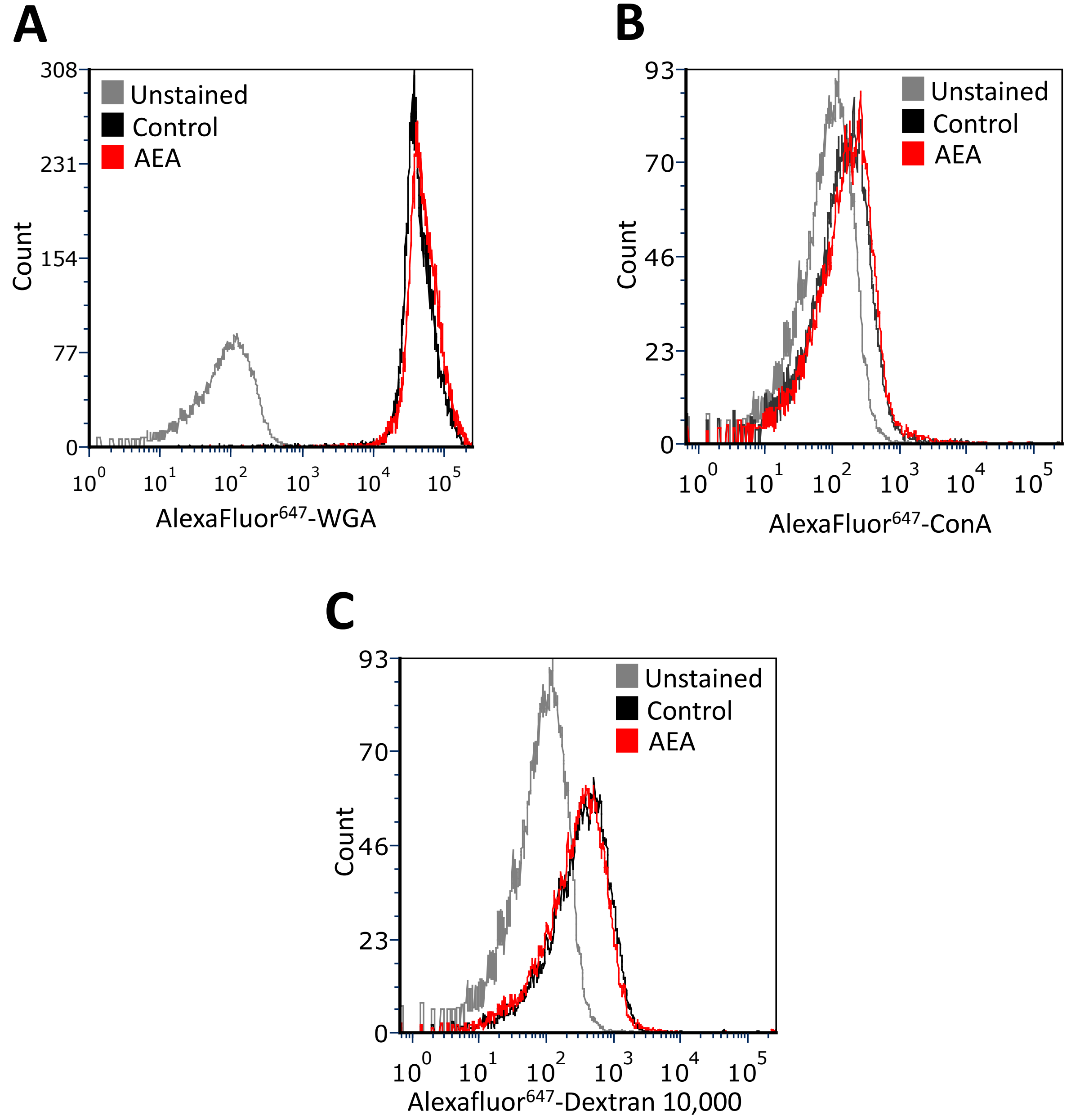


**Supplementary Figure S4. AEA did not alter the affinity of wheat germ agglutinin (WGA), concanavalin (ConA) or Dextran (average MW 10,000 Dalton) to the bacterial surface.** MDRSA CI-M was incubated in the absence or presence of 50 µg/ml of AEA for 2 h, and then the bacteria were incubated with 10 µg/ml of AlexaFluor$^{647}$-conjugated WGA. ConA or Dextran for 20 min prior to analysis by flow cytometry. Grey line: Unstained bacteria; Black line: Control; and Red line: AEA-treated bacteria.

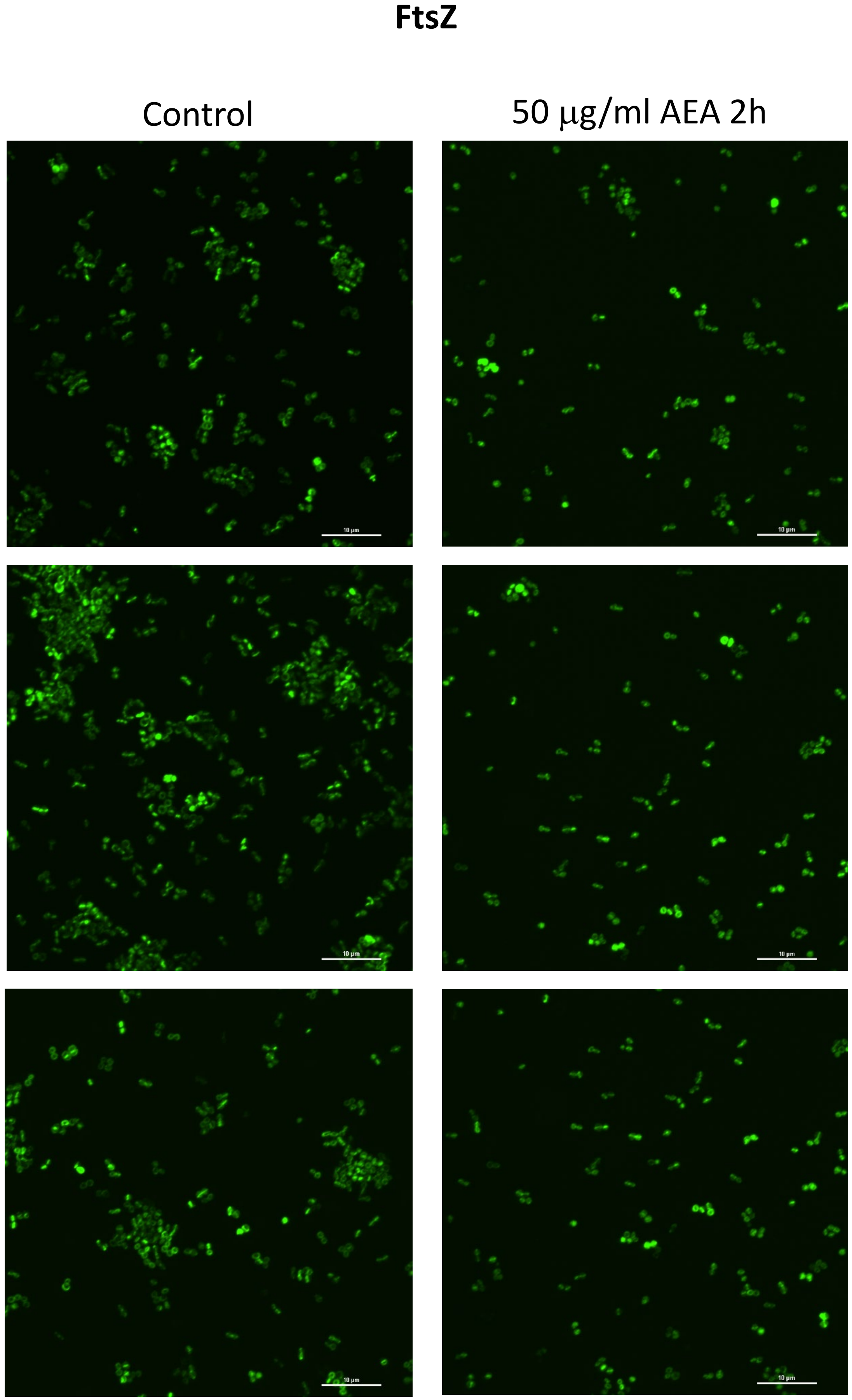


**Supplementary Figure S5.** LH607 expressing an inducible FtsZ-GFP (SA103; pLOW *ftsZ-gfp*, pGL485 (*erm*$^R$, *cat*$^R$, *tet*$^R$)) was exposed to 50 µM IPTG for 2 h and then incubated in the absence or presence of 50 µg/ml AEA for 2 h and the green fluorescence visualized by spinning disk confocal microscopy.

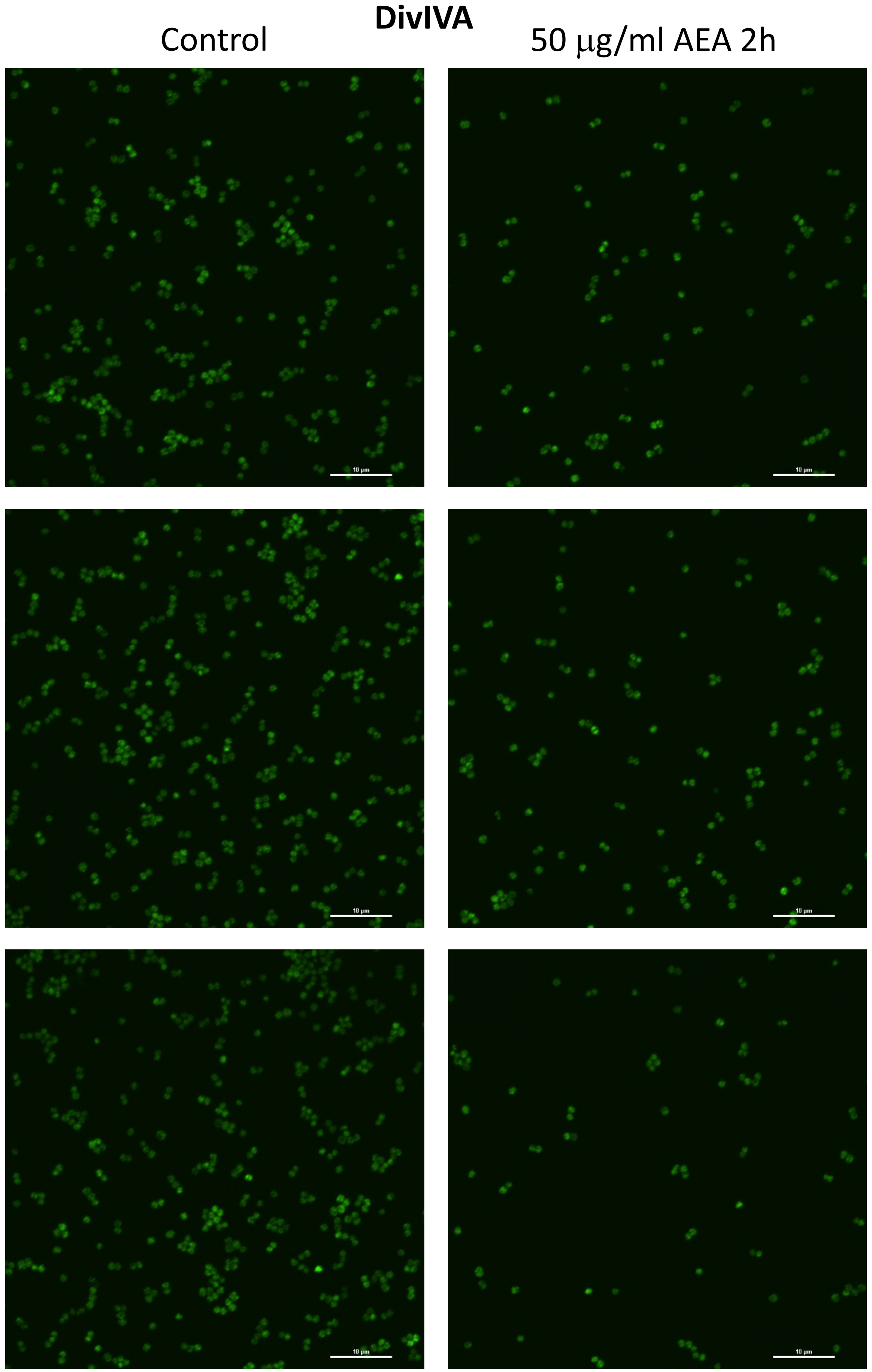


**Supplementary Figure S6.** LH607 expressing inducible DivIA-GFP (SA356; PdivIVA divIVA-gfp::Pspac divIVA, pGL485 (ermR catR tetR)) was exposed to 50 μM IPTG for 2 h and then incubated in the absence or presence of 50 μg/ml AEA for 2 h and the green fluorescence visualized by spinning disk microscopy.

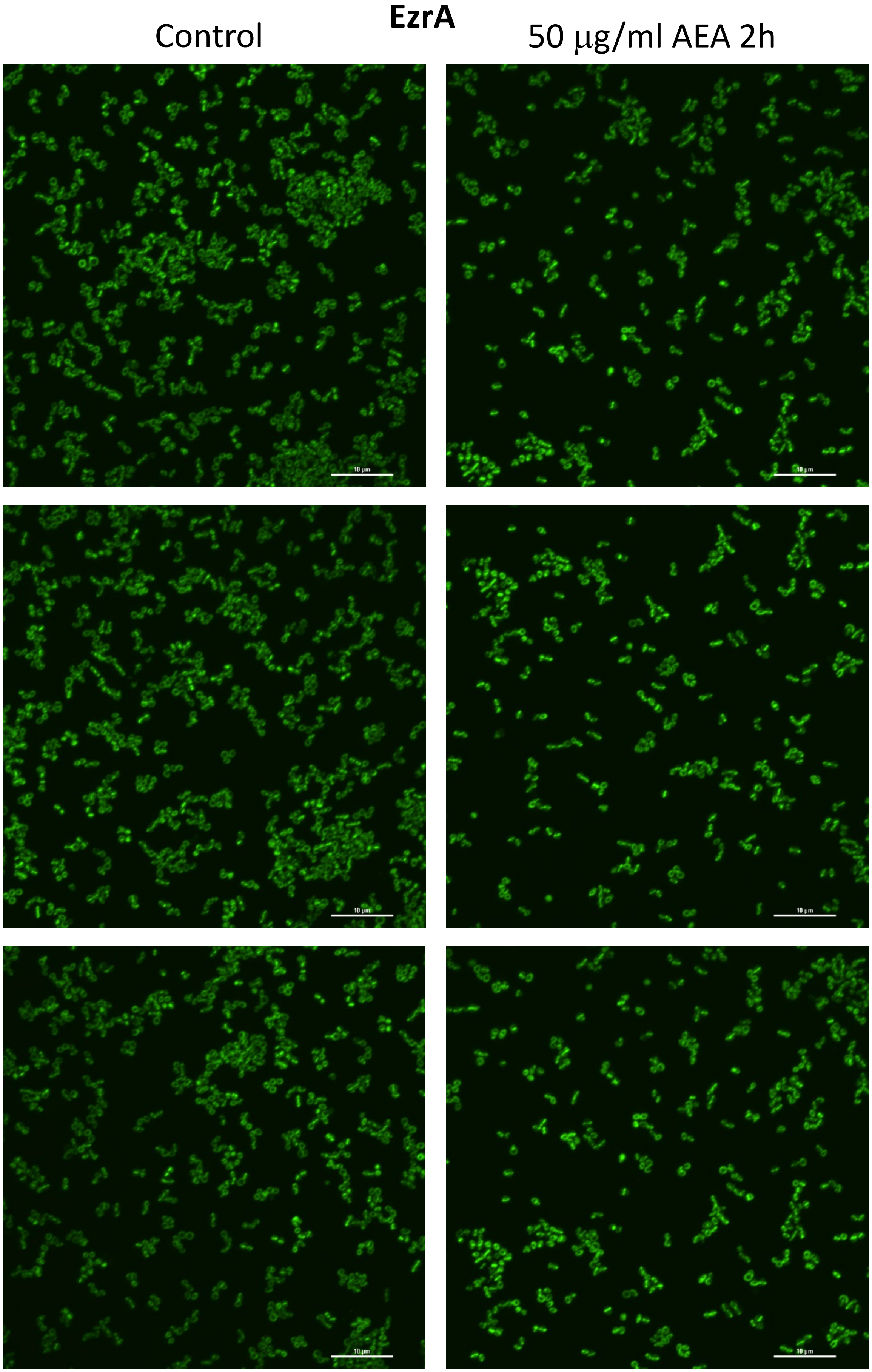


**Supplementary Figure S7.** LH607 expressing EzrA-GFP (SA353; *ezrA::ezrA-gfp*, pGL485 (*erm*$^{R}$ *cat*$^{R}$)) was exposed to 50 µM IPTG for 2 h and then incubated in the absence or presence of 50 µg/ml AEA for 2 h and the green fluorescence visualized by spinning disk microscopy.

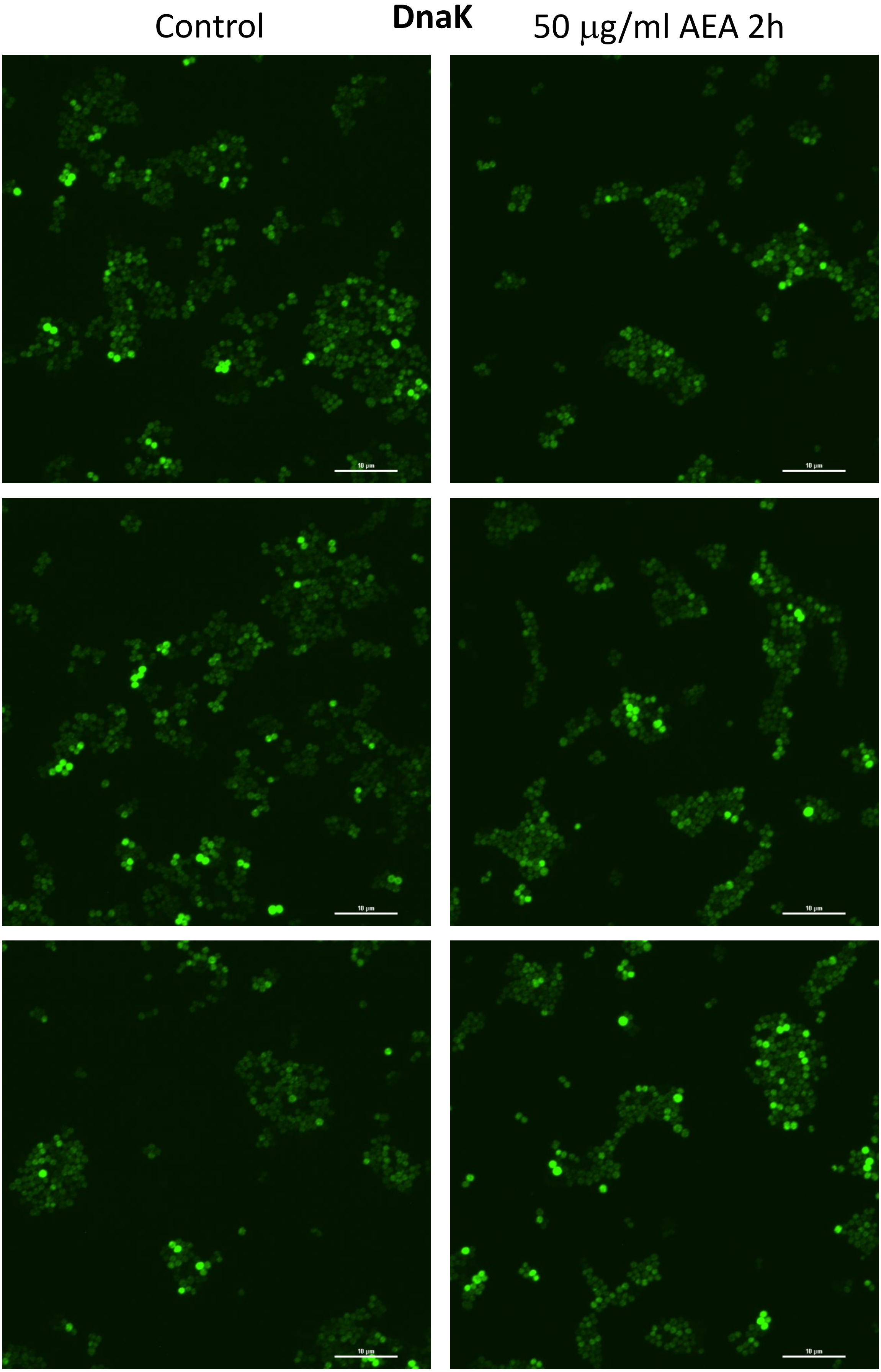


**Supplementary Figure S8.** RN4220 expressing inducible DnaK-GFP (SA307; pLOW *dnaK-msgfp*, pGL485 (*erm*$^R$ *cat*$^R$)) was exposed to 50 µM IPTG for 2 h and then incubated in the absence or presence of 50 µg/ml AEA for 2 h and the green fluorescence visualized by spinning disk microscopy.

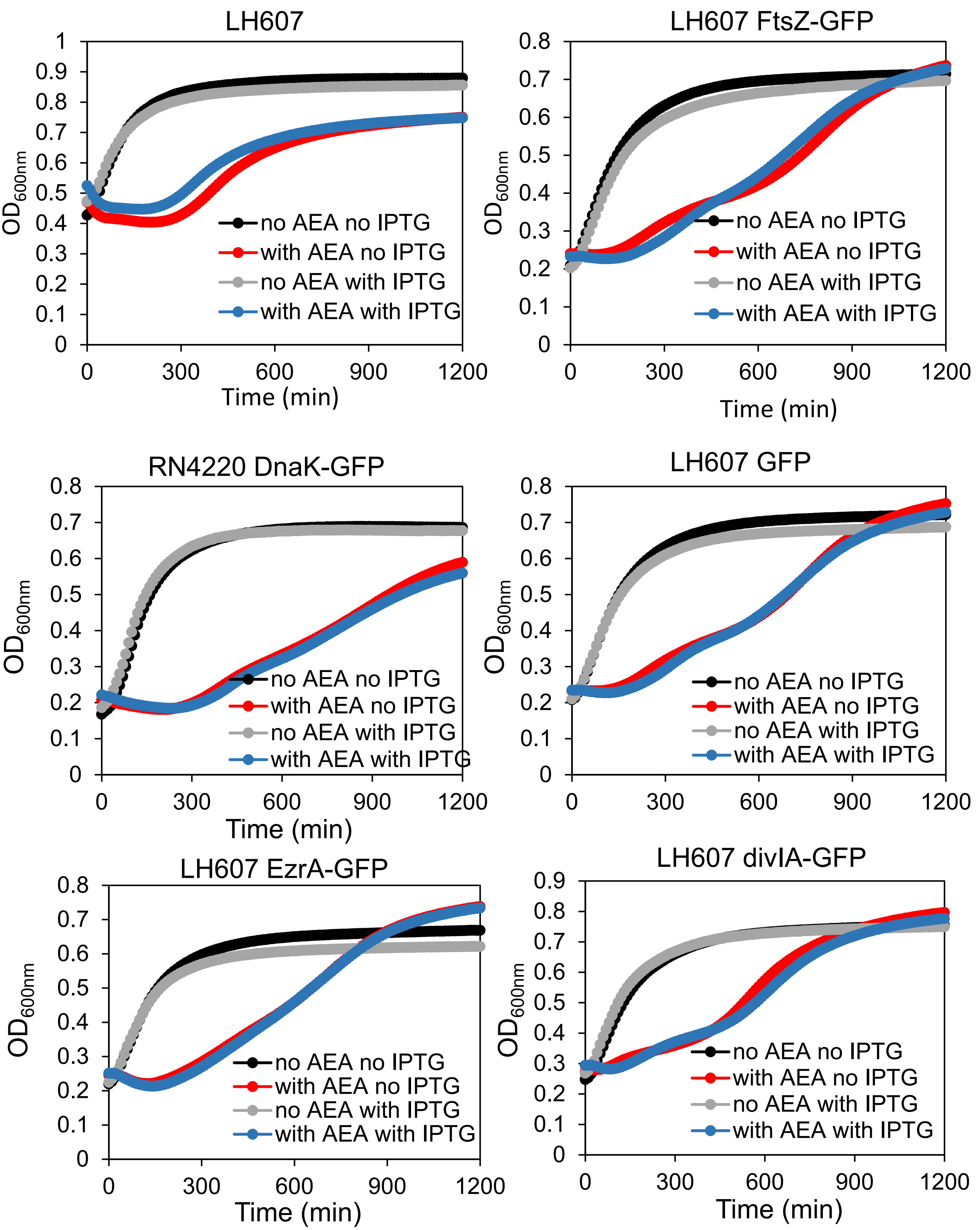


**Supplementary Figure S9.** Overnight cultures of the various *S. aureus* strains were
resuspended to an $OD_{600nm}$ of 0.1 in TSBG and incubated in TSBG for 2 hrs with 50 µM IPTG to induce gene expression. Then the bacteria were incubated in the absence or presence of 50 µg/ml AEA and the planktonic growth was measured each 10 min for 20 hrs.

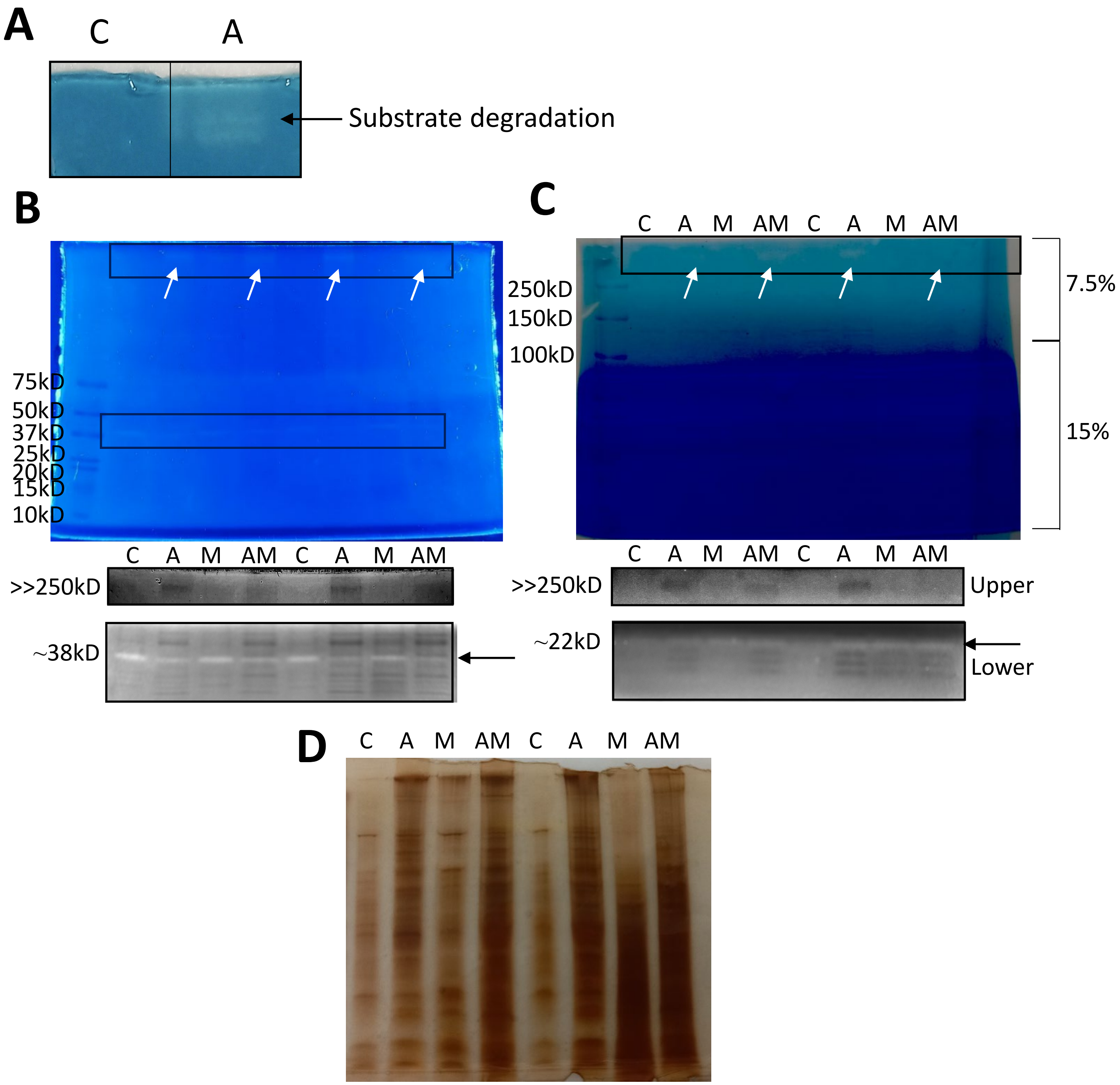


**Supplementary Figure S10. Appearance of a high molecular weight protease activity following AEA treatment. A. Gelatin zymogram:** SDS-extracts of control or 50 µg/ml AEA (2h)-treated MDRSA CI-M were run in a 5% non-reduced SDS-PAGE containing 1.2 mg/ml gelatin, and following incubation in protease reaction buffer for 24 h at 37°C, the gel was stained with Coomassie blue. A double band clear region was observed in the AEA-treated bacteria in the upper part of the gel representing protein complexes >>250kD. **B-C.** Gelatin (**B**) and casein (**C**) Zymograms of MDRSA CI-M that have been exposed to 50 µg/ml AEA and/or 50 µg/m MET for 2h. The upper part of the gel was 7.5%, while the lower part was 15%. Some of the substrate have electrophoresed from the 7.5% gel into the 15% gel. The two lower panels of B and C are black and white images of the two separate parts of the gel. To better visualize the >>250kD proteolytic bands, the images were inverted. In the lower panel, the proteolytic bands appear as clear regions within the gel. **D.** Silver stain of the same samples run in **B** and **C** using a 4-15% gradient gel. C=Control; A=AEA; M=MET; AM= AEA+MET. SDS-extraction was done on the same OD of bacteria.

The image of the whole gel of Suppl. Fig. 10A.

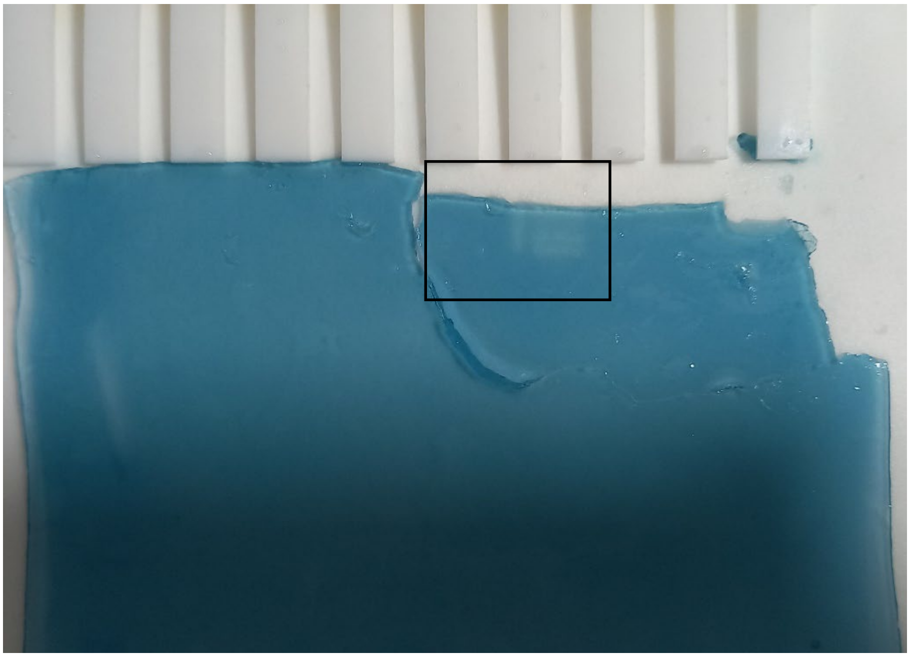

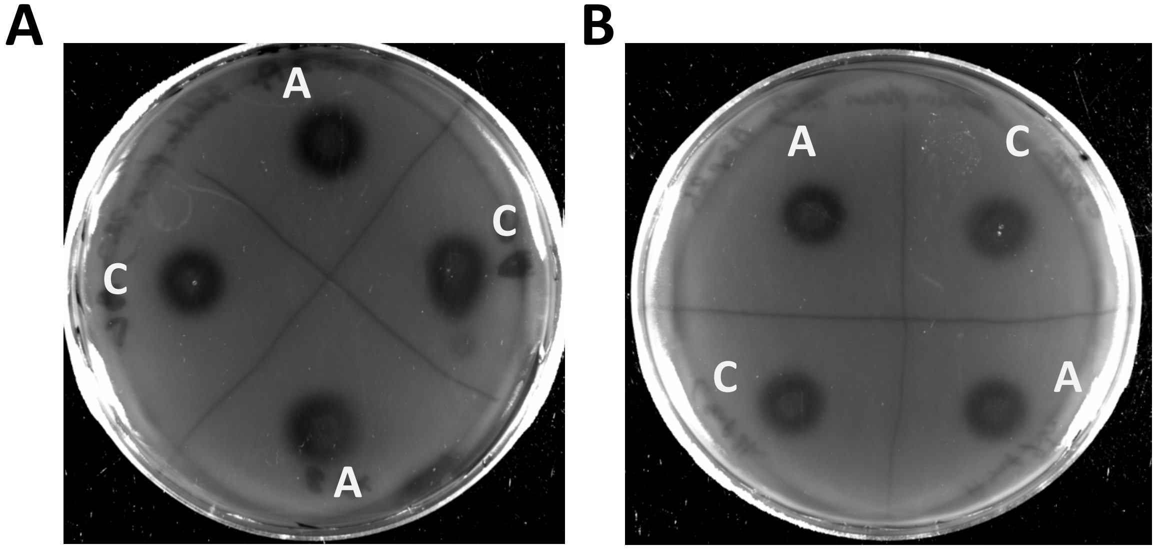


**Supplementary Figure S11. A. Anandamide treatment didn't interfere with secreted protease activity. A-B.** MDRSA CI-M was incubated in the absence or presence of 50 µg/ml AEA for 2 h, and then either 10 µl of the bacterial culture (**A**) or 10 µl of the culture supernatant (**B**) were inoculated on TSA agar plates containing 1.5% gelatin. The plates were incubated for 24 h and stained with crystal violet. Clear areas are seen in both control and AEA-treated bacteria indicating the degradation of gelatin by secreted proteases.

## Supplementary Table S1 –Relevant function of genes studied.

| Gene | Relevant gene functions | Reference |
|---|---|---|
| *abcA* | An ATP-dependent transporter that confers resistance to β-lactam antibiotics.<br>*abcA* is regulated by MgrA, NorG, Rot and sarZ. | [1,2] |
| *airSR* | AirSR two-component system is involved in resistance to reactive oxygen species by upregulating Staphyloxanthin production.<br>An *airSR Staphylococcus aureus* mutant exhibited reduced autolysis rates and reduced viability in the presence of vancomycin. | [3,4] |
| *arlR* | The two-component system ArlRS confers oxacillin resistance by inducing the expression of *spx*.<br>ArlR regulates the expression of more than hundred genes involved in different functions, including autolysis, cell division, growth, and pathogenesis.<br>ArlRS activates MgrA-mediated transcription of genes including cell wall-anchored adhesins (*ebh*, *sdrD*), polysaccharide and capsule synthesis genes, cell wall remodeling genes (*lytN*, *ddh*), genes involved in metal transport (*feoA*, *mntH*, *sirA*), anaerobic metabolism genes (*adhE*, *pflA*, *nrdDG*) and a large number of virulence factors (*lukSF*, *lukAB*, *nuc*, *gehB*, *norB*, *chs*, *scn* and *esxA*). | [5-8] |
| *atlA* | The initial attachment of *S. aureus* to a surface depends on the autolysin AtlA. Autolysins promotes the release of extracellular DNA to the biofilm matrix.<br>AtlA is also involved in cell division, cell wall turnover and bacterial lysis. | [9-11] |
| *cidA* | The CidA murein hydrolase regulator contributes to extracellular DNA release and biofilm formation in *Staphylococcus aureus*.<br>CidA increases the activity of murein hydrolases and promotes the detachment of bacteria from the biofilm and their spread to new infection sites in the dispersion phase.<br>CidA is a holin that is antagonized by the anti-holin Lrg system. | [12,13] |
| *clpP* | Inactivation of ClpXP protease led to increased β-lactam resistance in a MRSA USA300 strain.<br>A truncating mutation in *clpP* leads to vancomycin resistance.<br>ClpP degrades the autolysin Sle1 required for proper cell division.<br>ClpXP degrades superoxide dismutase (SodA) making the bacteria more prone to cell death.<br>ClpXP degrades the transcription factor Spx that confers antibiotic resistance. | [14-18] |
| *codY* | CodY is a nutrient-sensing regulator that affect the expression of over 200 genes. Among others it represses metabolic genes and virulence genes.<br>CodY represses capsule production.<br>A *codY* mutant is resistant to butyrate-induced growth inhibition.<br>A strain lacking *codY* regulatory activity produces a PIA-dependent biofilm. | [19-24] |
| *essC* | *essC* encodes a ESAT-6 secretion system C component belonging to the Type VII protein secretion system (T7SS) involved in virulence,<br>EssC possesses a membrane-bound multidomain ATPase and is involved in protein transport. | [25] |
| *fmhB* | FmhB is involved in the first step of peptidoglycan pentaglycine interpeptide formation. This interpeptide plays a role in the stability of the *S. aureus* cell wall, acts as an anchor for cell wall-associated proteins and is essential for methicillin resistance.<br>Any shortening of the pentaglycine side chain reduces or even abolishes methicillin resistance. | [26] |
| *gpsB* | GpsB localizes to mid-cell during cell division and interacts with the core divisome component FtsZ.<br>GpsB stimulates the GTPase activity of FtsZ and promotes bundling of FtsZ filaments, thus enabling cell division.<br>Depletion of GpsB caused cell division arrest and cell lysis, whereas overproduction of GpsB led to too early activation of FtsZ, resulting in the formation of enlarged cells. | [27] |

| | | |
|---|---|---|
| *isaA* | IsaA is a highly immunogenic, noncovalently cell wall-bound lytic transglycosylase that is co-regulated with the glycylglycine endopeptidase LytM.<br>Deletion of *isaA* in a MRSA strain led to decreased biofilm formation and reduced β-lactam resistance. | [28,29] |
| *lrgAB* | LrgA is an anti-holin that antagonizes the activity of murein hydrolases.<br>A *lrgAB* mutant showed increased extracellular murein hydrolase activity**.**<br>The *lrgAB* mutation were more sensitive to penicillin when approaching the stationary phase of growth, the time at which the *lrgAB* operon is maximally expressed. However, the *lrgAB* mutation did not affect penicillin-induced killing of cells growing in early-exponential phase, a time in which *lrgAB* expression is minimal.<br>Inactivation of *lrgB* increases cell lysis-dependent eDNA release and enhances biofilm formation. | [13,30,31] |
| *luxS* | LuxS mutants of *S. aureus* showed increased biofilm formation, reduced autolysis and increased expression of the vancomycin resistance-associated VraRS two-component regulatory system. | [32-35] |
| *lytSR* | LytSR senses changes in the membrane potential and confers resistance to antimicrobial peptides.<br>LytSR is a two-component system that regulates the expression of the anti-holin *lrgA* and *lrgB*. | [30,36,37] |
| *mecA* | mecA encodes for the PBP2a variant that shows low affinity for β-lactam antibiotics, and thus confers β-lactam resistance. | [38] |
| *pbp4* | A penicillin-binding protein that can confer β-lactam resistance, which is thought to be due to its high transpeptidase activity, that results in the production of a highly cross-linked cell wall peptidoglycan. | [1,39,40] |
| *prsA* | The foldase PrsA is required for proper folding of PBP2a and thereby promotes β-lactam resistance.<br>Deletion of *prsA* altered oxacillin resistance and caused a decrease in PBP2A membrane expression without affecting *mecA* mRNA levels. | [41,42] |
| *saeRS* | The SaeRS two component system controls the production of over 20 virulence factors including hemolysins, leukocidins, superantigens, surface proteins, and proteases.<br>SaeRS negatively regulates the expression of genes involved in cytolysis (*lrgA*) and autolysis (*lytS, atlE and aae*).<br>A *saeRS* mutant showed increase susceptibility to penicillin and oxacillin and was more prone to autolysis. | [43-45] |
| *sasG* | The SasG surface protein promotes biofilm formation, especially during the accumulation phase, which requires physiological levels of zinc ions. | [46,47] |
| *sigB* | SigB affects biofilm maturation by repressing the expression of RNAIII that has anti-biofilm activities.<br>A *sigB* mutant showed increased RNAIII expression, elevated extracellular protease levels and altered murine hydrolase activity. | [48] |
| *sle1* | The autolysin Sle1 is important for the onset of daughter cell separation.<br>Sle1 is a substrate of the ClpXP protease.<br>High Sle1 levels in bacteria lacking ClpXP activity confer β-lactam hyper-resistance. | [18] |
| *sprX* | SprX is a small non-coding RNA that positively regulates the expression of the autolysin regulator WalR, resulting in increased induction of the autolysins *isaA* and *lytM*.<br>SprX upregulates the expression of the virulence genes cell wall-associated clumping factor B (*clfB*) and delta hemolysin (*hld*).<br>Down-regulation of *sprX* resulted in decreased biofilm formation and higher resistance to Triton X-100-induced lysis. | [49,50] |
| *spx* | Spx is a stress-induced transcriptional regulator that controls the expression of *trfA* implicated in antibiotic resistance.<br>Spx expression is regulated by the ArlRS two-component system.<br>Deletion of *arlRS* sensitized MRSA to oxacillin, while overexpression of Spx in the *ΔarlRS* strain restored oxacillin resistance. | [5,51-53] |

| | | |
|---|---|---|
| | A *spx* mutant was hypersensitive to a wide range of stress conditions including high and low temperature, high osmolarity, and hydrogen peroxide due to lack of *trxB* thioredoxin reductase transcription.<br>YjbH controls the degradation of Spx by ClpP. | |
| *tarO* | TarO is involved in the initial step of cell wall teichoic acid synthesis. TagO catalyzes the reversible transfer of GlcNAc-1-P from UDP–GlcNAc to the undecaprenyl phosphate scaffold to produce lipid-α (GlcNAcα–PP-Undecaprenyl).<br>A *tagO* mutant showed increased cell surface hydrophobicity, enhanced autolytic activity, impaired biofilm formation, and reduced expression of *icaADBC* and *PIA* genes.<br>Deletion of *tarO* in a MRSA strain restored their sensitivity to methicillin. | [54-56] |
| *tarA* | TagA is involved in the step after TagO in cell wall teichoic acid synthesis. TagA is a ManNAc transferase that adds ManNAc from a sugar nucleotide donor (UDP-ManNAc), producing a ManNAc (β1 → 4) GlcNAcα–PP- Undecaprenyl product, called lipid-β. | [54] |
| *tarM/tarS* | TarM and TarS add α-linked and β-linked N-acetylglucosamine, respectively, to the poly-ribitol chain of the growing wall teichoic acid.<br>Eliminating *tarS* from a MRSA strain sensitized the bacteria to β-lactams. This suggests that β-O-GlcNAcylation of wall teichoic acids is required for MRSA resistance. | [54,57] |
| *tarG/tarH* | The TagGH transporter transfer the wall teichoic acid across the membrane.<br>TagG is essential for bile-induced biofilm formation in *S. aureus* and its expression protects the bacteria from bile-induced cell lysis. | [54,58] |
| *trfA* | TrfA is required for the degradation of the MazE antitoxin and thus affects dormancy and tolerance to antibiotics.<br>*trfA* transcription is regulated by the redox sensitive transcriptional factor Spx. | [51,53,59] |
| *walKR* | The WalKR two-component system controls cell wall metabolism by regulating autolysin production such as *sceD*, *ssaA*, *lytM* and *atlA*.<br>A deletion mutation in *walRK* conferred vancomycin resistance. | [15,60] |

**Supplementary Table S2 – Primers used for quantitative real-time PCR for *Staphylococcus aureus*.**

| Gene | Forward Primer | Reverse Primer | Reference |
|---|---|---|---|
| 16S rRNA | CCAGCAGCCGCGGTAAT | CGCGCTTTACGCCCAATA | [61] |
| *abcA* | CAAGAACCTATTGAACCGACAGAA | GTGGGATTTGGAACGACACA | [1] |
| *airR* | TGCTGATGGTTATGAAATGA | CATCTTGTGCCTTAGGATGT | [19] |
| *airS* | TTCCTAGCCAAAATGACAATA | TTCAGTATTTGGAGACGCTAC | [19] |
| *arlR* | TTCTTCAATATCAAACGGCTTA | GACAACAATCTACACCTAT | [5] |
| *asnC* | TCGGTGGATCTGAACGTGTGGA | GTGGCACACTACCATAACGACG | [62] |
| *atlA* | AACAGCACCAACGGATTAC | CATAGTCAGCATAGTTATTCATTG | [49] |
| *cidA* | CTACTACTACAACTAGGAATCATC | TTTAGCGTAATTTCGGAAGC | [63] |
| *clpP* | AACAACAAATCGCGGTGAAC | CATAAATCGCAAAACCAGCTGT | This paper |
| *codY* | ATCGCATCAAAAGTTGCAGA | CGTGATTCAATTACACCAGCA | [19] |
| *essC* | ACCATCGTTCGCCAAGGA | TGGCTGTGGCGGTCTTTC | [64] |
| *fmhB* | AAGCGAGGTACGACAGTAGAACG | CATCTCCATCTTCATGCAACGCA | [61] |
| *glyA* | CTACAAACTCACAGCCAC | GTATCGGAAGCGGTTATG | [61] |
| *gmk2* | CCATCTGGAGTAGGTAAAGG | CTACGCCATCAACTTCAC | [61] |
| *gtf* | TGGTGACGCCGAAGGACTC | GCAGCACGAGCAGGAACAC | [61] |
| *gpsB* | TCCTGAGGTCTTGATGTTGC | TGGCTCGTGGCTATAGAAGA | This paper |
| *gyrA* | TGGCCCAAGACTTTAGTTATCGTTATCC | TGGGGAGGAATATTTGTAGCCATACCTAC | [61] |
| *gyrB* | GGTGCTGGGCAAATACAAGT | TCCCACACTAAATGGTGCAA | [61] |
| *isaA* | GCAGGTGCTACTGGTTCATCAG | GATTCACGAGCGATGATTGC | [49] |
| *lrgA* | TGAAACAACAAAAAGACGCATCAAAACCAG | ACTTCGCCTAACTTAACAGCACCAG | [6] |
| *lrgB* | TATTTGGTGTGGCCTTCCTC | AAACAGATTGTTGCCGGTTC | [63] |
| *luxS* | CGGACTACATTCATTAGAACATT | TTACAAGCAGGCACTTCA | [65] |
| *lytR* | ATTAGGAGCTAAGATTCAAAAGATG | TTGACTGCTTGTTCAATACG | [63] |
| *lytS* | GCATGGTTCTATCGTCGGTACATTG | ACTTACTTTGCGTTTCGGCTTCAC | [6] |
| *pbp4* | CTAAAGGTGAGCAAAGGATAAATGG | TCTCTTGGATAGTCCGCGTGT | [1] |
| *proC* | GGCAGGTATTCCGATTGA | CCAGTAACAGAGTGTCCAAC | [61] |
| *prsA* | AGTTAATGATAAGAAGATTGACGAACAAA | GAAGGGCCTTTTCAAATTTATCTTT | [42] |
| *recF* | AGTTATAGACACGGCACG | GCGTCGTCTTATTTGAGG | [61] |
| *rho* | GGAAGATACGACGTTCAGAC | GAAGCGGGTGGAAGTTTA | [61] |
| *RNAII* | TATGAATAAATGCGCTGATGATATACCACG | TTTTAAAGTTGATAGACCTAAACCACGACC | [61] |
| *rpoB* | TCCTGTTGAACGCGCATGTAA | GCTGGTATGGCTCGTGATGGTA | [61] |
| *saeR* | AAGTGGCGACCATTACAT | CATTATTGCCTCAAATACGT | [66] |
| *saeS* | TGCCAATACCTTCATCGCTAA | CAATATCGAACGCCACTTGA | [67] |
| *sasG* | ATCGTCAGTCACTCATAAC | TATCAACACTTCCGTAACC | [65] |
| *sigB* | TCGATAACTATAACCAAAGCCT | AAGTGATTCGTAAGGACGTCT | [68] |
| *sle1* | TCAGGATCTGCAACAACGAC | CCTTTACCAATTTCAGCACGAC | [15] |
| *sprX* | ATAATCTTTCTAGACGTATTCAAA | CAGGCTATATAGTTCACTCCTACT | [6] |
| *spx* | GCTTATTACGTCGTCCAATTATTTTA | CGTACGAACTTTTCTAGGTAAGAA | [16] |
| *tarA* | GTTGCTGATGGGACAGGAGT | TGCATATTGTGCCGCTTCTA | [17] |
| *tarG* | ATCAGTATGTGGTTCTTCATC | TGCTGCACGGTATGATTCAG | [17] |
| *tarH* | ATCATTGGCGGTTCTTTGTC | TGCACGCATACCACTTGAAT | [17] |
| *tarO* | TTCCATCCTGCCAAAATA | GAATGGAACTGCTAAGATAACA | [17] |
| *tarM* | TAATGCTAATAATGGTGCTG | GGTCCATCACAAATCATAAT | [18] |
| *tarS* | CACGAAACAAGAAGCACA | TGATTACCAACACGCACT | [18] |
| *trfA* | ATCGAGGCCCGTGGATTTAG | TCGACACCTTTTTCAAAGGCA | [4] |
| *walR* | CAAATGGCTAGAAAAGTTGTTGTAG | CAGTAAGCATTATTATTGGCATTTCG | [6] |

## References


1. Banerjee, S.; Sionov, R.V., Feldman. M.; Smoum, R.; Mechoulam, R.; Steinberg, D. Anandamide alters the membrane properties, halts the cell division and prevents drug efflux in multidrug resistant *Staphylococcus aureus*. *Sci Rep* **2021**, *11*, 8690.
2. Villet, R.A.; Truong-Bolduc, Q.C.; Wang, Y.; Estabrooks, Z.; Medeiros, H.; Hooper, D.C. Regulation of expression of *abcA* and its response to environmental conditions. *J Bacteriol* **2014**, *196*, 1532-1539.
3. Batte, J.L.; Sahukhal, G.S.; Elasri, M.O. MsaB and CodY interact to regulate *Staphylococcus aureus* capsule in a nutrient-dependent manner. *J Bacteriol* **2018**, *200*, e00294-18.
4. Bai, J.; Zhu, X.; Zhao, K.; Yan, Y.; Xu, T.; Wang, J.; Zheng, J.; Huang, W.; Shi, L.; Shang, Y.; Lv, Z.; Wang, X.; Wu, Y.; Qu, D. The role of ArlRS in regulating oxacillin susceptibility in methicillin-resistant *Staphylococcus aureus* indicates it is a potential target for antimicrobial resistance breakers. *Emerg Microbes Infect* **2019**, *8*, 503-515.
5. Chung, P.Y.; Chung, L.Y.; Navaratnam, P. Transcriptional profiles of the response of methicillin-resistant *Staphylococcus aureus* to pentacyclic triterpenoids. *PLoS One* **2013**, *8*, e56687.
6. Buchad, H.; Nair, M. The small RNA SprX regulates the autolysin regulator WalR in *Staphylococcus aureus*. *Microbiol Res* **2021**, *250*, 126785.
7. Neumann, Y.; Ohlsen, K.; Donat, S.; Engelmann, S.; Kusch, H.; Albrecht, D.; Cartron, M.; Hurd, A.; Foster, S.J. The effect of skin fatty acids on *Staphylococcus aureus*. *Arch Microbiol* **2015**, *197*, 245-267.
8. Ishii, K.; Adachi, T.; Yasukawa, J.; Suzuki, Y.; Hamamoto, H.; Sekimizu, K. Induction of virulence gene expression in *Staphylococcus aureus* by pulmonary surfactant. *Infect Immun* **2014**, *82*, 1500-1510.
9. Liang, X.; Zheng, L.; Landwehr, C.; Lunsford, D.; Holmes, D.; Ji, Y. Global regulation of gene expression by ArlRS, a two-component signal transduction regulatory system of *Staphylococcus aureus*. *J Bacteriol* **2005**, *187*, 5486-5492.
10. Zheng, J.; Shang, Y.; Wu, Y.; Wu, J.; Chen, J.; Wang, Z.; Sun, X.; Xu, G.; Deng, Q.; Qu, D.; Yu, Z. Diclazuril inhibits biofilm formation and hemolysis of *Staphylococcus aureus*. *ACS Infect Dis* **2021**, *7*, 1690-1701.
11. Jousselin, A.; Manzano, C.; Biette, A.; Reed, P.; Pinho, M.G.; Rosato, A.E.; Kelley, W.L.; Renzoni, A. The *Staphylococcus aureus* chaperone PrsA is a new auxiliary factor of oxacillin resistance affecting Penicillin-Binding Protein 2A. *Antimicrob Agents Chemother* **2015**, *60*, 1656-1666.
12. Salaheen, S.; Peng, M.; Joo, J.; Teramoto, H.; Biswas, D. Eradication and sensitization of methicillin resistant *Staphylococcus aureus* to methicillin with bioactive extracts of berry pomace. *Front Microbiol* **2017**, *8*, 253.
13. Sethupathy, S.; Vigneshwari, L.; Valliammai, A.; Balamurugan, K.; Pandian, S.K. L-Ascorbyl 2, 6-Dipalmitate inhibits biofilm and virulence in methicillin-resistant *Staphylococcus aureus* and prevents triacylglyceride accumulation in *Caenorhabditis elegans*. *RSC Adv* **2017**, *7*, 23392-23406.
14. Kannappan, A.; Srinivasan, R.; Nivetha, A.; Annapoorani, A.; Pandian, S.K.; Ravi, A.V. Anti-virulence potential of 2-hydroxy-4-methoxybenzaldehyde against methicillin-resistant *Staphylococcus aureus* and its clinical isolates. *Appl Microbiol Biotechnol* **2019**, *103*, 6747-6758.
15. Wassmann, C.S.; Højrup, P.; Klitgaard, J.K. Cannabidiol is an effective helper compound in combination with bacitracin to kill Gram-positive bacteria. *Sci Rep* **2020**, *10*, 4112.
16. Jousselin, A.; Kelley, W.L.; Barras, C.; Lew, D.P.; Renzoni, A. The *Staphylococcus aureus* thiol/oxidative stress global regulator Spx controls *trfA*, a gene implicated in cell wall antibiotic resistance. *Antimicrob Agents Chemother* **2013**, *57*, 3283-3292.
17. Wanner, S.; Schade, J.; Keinhörster, D.; Weller, N.; George, S.E.; Kull, L.; Bauer, J.; Grau, T.; Winstel, V.; Stoy, H.; Kretschmer, D.; Kolata, J.; Wolz, C.; Bröker, B.M.; Weidenmaier, C. Wall teichoic acids mediate increased virulence in *Staphylococcus aureus*. *Nat Microbiol* **2017**, *2*, 16257.
18. Winstel, V.; Kühner, P.; Salomon, F.; Larsen, J.; Skov, R.; Hoffmann, W.; Peschel, A.; Weidenmaier, C. Wall teichoic acid glycosylation governs *Staphylococcus aureus* nasal colonization. *mBio* 2015, *6*, e00632.
19. Truong-Bolduc, Q.C.; Hooper, D.C. The transcriptional regulators NorG and MgrA modulate resistance to both quinolones and beta-lactams in *Staphylococcus aureus*. *J Bacteriol* **2007**, *189*, 2996-3005.
20. Hall, J.W.; Yang, J.; Guo, H.; Ji, Y. The *Staphylococcus aureus* AirSR Two-component system mediates reactive oxygen species resistance via transcriptional regulation of Staphyloxanthin production. *Infect Immun* **2017**, *85*, e00838-16.
21. Sun, H.; Yang, Y.; Xue, T.; Sun, B. Modulation of cell wall synthesis and susceptibility to vancomycin by the two-component system AirSR in *Staphylococcus aureus* NCTC8325. *BMC Microbiol* **2013**, *13*, 286.

22. Crosby, H.A.; Tiwari, N.; Kwiecinski, J.M.; Xu, Z.; Dykstra, A.; Jenul, C.;, Fuentes, E.J. Horswill, A.R. The *Staphylococcus aureus* ArlRS two-component system regulates virulence factor expression through MgrA. *Mol Microbiol* **2020**, *113*, 103-122.
23. Fournier, B.; Klier, A.; Rapoport, G. The two-component system ArlS-ArlR is a regulator of virulence gene expression in *Staphylococcus aureus*. *Mol Microbiol* **2001**, *41*, 247-261.
24. Heilmann, C.; Hussain, M.; Peters, G.; Götz, F. Evidence for autolysin-mediated primary attachment of *Staphylococcus epidermidis* to a polystyrene surface. *Mol Microbiol* **1997**, *24*, 1013-1024.
25. Houston, P.; Rowe. S.E.; Pozzi, C.; Waters, E.M.; O'Gara, J.P. Essential role for the major autolysin in the fibronectin-binding protein-mediated *Staphylococcus aureus* biofilm phenotype. *Infect Immun* **2011**, *79*, 1153-1165.
26. Bose, J.L.; Lehman, M.K.; Fey, P.D.; Bayles, K.W. Contribution of the *Staphylococcus aureus* Atl AM and GL murein hydrolase activities in cell division, autolysis, and biofilm formation. *PLoS One* **2012**, *7*, e42244.
27. Rice, K.C.; Mann, E.E.; Endres, J.L.; Weiss, E.C.; Cassat, J.E.; Smeltzer, M.S.; Bayles, K.W. The *cidA* murein hydrolase regulator contributes to DNA release and biofilm development in *Staphylococcus aureus*. *Proc Natl Acad Sci U S A* **2007**, *104*, 8113-8118.
28. Ranjit, D.K.; Endres, J.L.; Bayles, K.W. *Staphylococcus aureus* CidA and LrgA proteins exhibit holin-like properties. *J Bacteriol* **2011**, *193*, 2468-2476.
29. Bæk, K.T.; Gründling, A.; Mogensen, R.G.; Thøgersen, L.; Petersen, A.; Paulander, W.; Frees, D. β-Lactam resistance in methicillin-resistant *Staphylococcus aureus* USA300 is increased by inactivation of the ClpXP protease. *Antimicrob Agents Chemother* **2014**, **58**, 4593-4603.
30. Shoji, M.; Cui, L.; Iizuka, R.; Komoto, A.; Neoh, H.M.; Watanabe, Y.; Hishinuma, T.; Hiramatsu, K. *walK* and *clpP* mutations confer reduced vancomycin susceptibility in *Staphylococcus aureus*. *Antimicrob Agents Chemother* **2011**;**55**:3870-3881.
31. Feng, J.; Michalik, S.; Varming, A.N.; Andersen, J.H.; Albrecht, D.; Jelsbak, L.; Krieger, S.; Ohlsen, K.; Hecker, M.; Gerth, U.; Ingmer, H.; Frees, D. Trapping and proteomic identification of cellular substrates of the ClpP protease in *Staphylococcus aureus*. *J Proteome Res* **2013**, *12*, 547-558.
32. Alqarzaee, A.A.; Chaudhari, S.S.; Islam, M.M.; Kumar, V.; Zimmerman, M.C.; Saha, R.; Bayles, K.W.; Frees, D.; Thomas, V.C. Staphylococcal ClpXP protease targets the cellular antioxidant system to eliminate fitness-compromised cells in stationary phase. *Proc Natl Acad Sci U S A* **2021**, *118*, e2109671118.
33. Thalsø-Madsen, I.; Torrubia, F.R.; Xu, L.; Petersen, A.; Jensen, C.; Frees, D. The Sle1 cell wall amidase is essential for β-Lactam resistance in community-acquired methicillin-resistant *Staphylococcus aureus* USA300. *Antimicrob Agents Chemother* **2019**, *64*, e01931-19.
34. Fletcher, J.R.; Villareal, A.R.; Penningroth, M.R.; Hunter, R.C. *Staphylococcus aureus* overcomes anaerobe-derived short-chain fatty acid stress via FadX and the CodY regulon. *J Bacteriol* **2022**, e0006422.
35. Pohl, K.; Francois, P.; Stenz, L.; Schlink, F.; Geiger, T.; Herbert, S.; Goerke, C.; Schrenzel, J.; Wolz, C. CodY in *Staphylococcus aureus*: A regulatory link between metabolism and virulence gene expression. *J Bacteriol* **2009**, *191*, 2953-2963.
36. Majerczyk, C.D.; Sadykov, M.R.; Luong, T.T.; Lee, C.; Somerville, G.A.; Sonenshein, A.L. *Staphylococcus aureus* CodY negatively regulates virulence gene expression. *J Bacteriol* **2008**, *190*, 2257-2265.
37. Waters, N.R.; Samuels, D.J.; Behera, R.K.; Livny, J.; Rhee, K.Y.; Sadykov, M.R.; Brinsmade, S.R. A spectrum of CodY activities drives metabolic reorganization and virulence gene expression in *Staphylococcus aureus*. Mol Microbiol **2016**, *101*, 495-514.
38. Majerczyk, C.D.; Dunman, P.M.; Luong, T.T.; Lee, C.Y.; Sadykov, M.R.; Somerville, G.A.; Bodi, K.; Sonenshein, A.L. Direct targets of CodY in *Staphylococcus aureus*. *J Bacteriol* **2010**, *192*, 2861-2877.
39. Bowman, L.; Palmer, T. The Type VII Secretion System of *Staphylococcus*. *Annu Rev Microbiol* **2021**, *75*, 471-494.
40. Rohrer, S.; Ehlert, K.; Tschierske, M.; Labischinski, H.; Berger-Bächi, B. The essential *Staphylococcus aureus* gene *fmhB* is involved in the first step of peptidoglycan pentaglycine interpeptide formation. *Proc Natl Acad Sci U S A* **1999**, *96*, 9351-9356.
41. Eswara, P.J.; Brzozowski, R.S.; Viola, M.G.; Graham, G.; Spanoudis, C.; Trebino, C.; Jha, J.; Aubee, J.I.; Thompson, K.M.; Camberg, J.L.; Ramamurthi, K.S. An essential *Staphylococcus aureus* cell division protein directly regulates FtsZ dynamics. *Elife* **2018**, *7*, e38856.
42. Lopes, A.A.; Yoshii, Y.; Yamada, S.; Nagakura, M.; Kinjo, Y.; Mizunoe, Y.; Okuda, K.I. Roles of lytic transglycosylases in biofilm formation and β-lactam resistance in methicillin-resistant *Staphylococcus aureus*. *Antimicrob Agents Chemother* **2019**, *63*, e01277-19.

43. Stapleton, M.R.; Horsburgh, M.J.; Hayhurst, E.J.; Wright, L.; Jonsson, I.M.; Tarkowski, A.; Kokai-Kun, J.F.; Mond, J.J.; Foster, S.J. Characterization of IsaA and SceD, two putative lytic transglycosylases of *Staphylococcus aureus*. *J Bacteriol* **2007**, *189*, 7316-7325.
44. Groicher, K.H.; Firek, B.A.; Fujimoto, D.F.; Bayles, K.W. The *Staphylococcus aureus lrgAB* operon modulates murein hydrolase activity and penicillin tolerance. *J Bacteriol* **2000**, *182*, 1794-1801.
45. Beltrame, C.O.; Côrtes, M.F.; Bonelli, R.R.; Côrrea, A.B.; Botelho, A.M.; Américo, M.A.; Fracalanzza, S.E.; Figueiredo, A.M. Inactivation of the autolysis-related genes *lrgB* and *yycI* in *Staphylococcus aureus* increases cell lysis-dependent eDNA release and enhances biofilm development *in vitro* and *in vivo*. *PLoS One* **2015**, *10*, e0138924.
46. Yu, D.; Zhao, L.; Xue, T.; Sun, B. *Staphylococcus aureus* autoinducer-2 quorum sensing decreases biofilm formation in an icaR-dependent manner. *BMC Microbiol* **2012**, *12*, 288.
47. Xue, T.; Zhao, L.; Sun, B. LuxS/AI-2 system is involved in antibiotic susceptibility and autolysis in *Staphylococcus aureus* NCTC 8325. *Int J Antimicrob Agents* **2013**, *41*, 85-89.
48. Ma, R.; Qiu, S.; Jiang, Q.; Sun, H.; Xue, T.; Cai, G.; Sun, B. AI-2 quorum sensing negatively regulates *rbf* expression and biofilm formation in *Staphylococcus aureus*. *Int J Med Microbiol* **2017**, *307*, 257-267.
49. Zhao, L.; Xue, T.; Shang, F.; Sun, H.; Sun, B. *Staphylococcus aureus* AI-2 quorum sensing associates with the KdpDE two-component system to regulate capsular polysaccharide synthesis and virulence. *Infect Immun* **2010**, *78*, 3506-3515.
50. Yang, S.J.; Xiong, Y.Q.; Yeaman, M.R.; Bayles, K.W.; Abdelhady, W.; Bayer, A.S. Role of the LytSR two-component regulatory system in adaptation to cationic antimicrobial peptides in *Staphylococcus aureus*. *Antimicrob Agents Chemother* **2013**, *57*, 3875-3882.
51. Brunskill, E.W.; Bayles, K.W. Identification and molecular characterization of a putative regulatory locus that affects autolysis in *Staphylococcus aureus*. *J Bacteriol* **1996**, *178*, 611-618.
52. Peacock, S.J.; Paterson, G.K. Mechanisms of methicillin resistance in *Staphylococcus aureus*. *Annu Rev Biochem* **2015**, *84*, 577-601.
53. da Costa, T.M.; de Oliveira, C.R.; Chambers, H.F.; Chatterjee, S.S. PBP4: A new perspective on *Staphylococcus aureus* β-lactam resistance. *Microorganisms* **2018**, *6*, 57.
54. Hamilton, S.M.; Alexander, J.A.N.; Choo, E.J.; Basuino, L.; da Costa, T.M.; Severin, A.; Chung, M.; Aedo, S.; Strynadka, N.C.J.; Tomasz, A.; Chatterjee, S.S.; Chambers, H.F. High-level resistance of *Staphylococcus aureus* to β-Lactam antibiotics mediated by Penicillin-Binding Protein 4 (PBP4). *Antimicrob Agents Chemother* **2017**, *61*, e02727-16.
55. Roch, M.; Lelong, E.; Panasenko, O.O.; Sierra, R.; Renzoni, A.; Kelley, W.L. Thermosensitive PBP2a requires extracellular folding factors PrsA and HtrA1 for *Staphylococcus aureus* MRSA β-lactam resistance. *Commun Biol* **2019**, *2*, 417.
56. Liu, Q.; Yeo, W.S.; Bae, T. The SaeRS two-component system of *Staphylococcus aureus*. *Genes* (Basel) **2016**, *7*, 81.
57. Lou, Q.; Ma, Y.; Qu, D. Two-component signal transduction system SaeRS is involved in competence and penicillin susceptibility in *Staphylococcus epidermidis*. *J Basic Microbiol* **2016**, *56*, 358-368.
58. Lou, Q.; Zhu, T.; Hu, J.; Ben, H.; Yang, J.; Yu, F.; Liu, J.; Wu, Y.; Fischer, A.; Francois, P.; Schrenzel, J.; Qu, D. Role of the SaeRS two-component regulatory system in *Staphylococcus epidermidis* autolysis and biofilm formation. *BMC Microbiol* **2011**, *11*, 146.
59. Geoghegan, J.A.; Corrigan, R.M.; Gruszka, D.T.; Speziale, P.; O'Gara, J.P.; Potts, J.R.; Foster, T.J. Role of surface protein SasG in biofilm formation by *Staphylococcus aureus*. *J Bacteriol* **2010**, *192*, 5663-5673.
60. Formosa-Dague, C.; Speziale, P.; Foster, T.J.; Geoghegan, J.A.; Dufrêne, Y.F. Zinc-dependent mechanical properties of *Staphylococcus aureus* biofilm-forming surface protein SasG. *Proc Natl Acad Sci U S A* **2016**, *113*, 410-415.
61. Lauderdale, K.J.; Boles, B.R.; Cheung, A.L.; Horswill, A.R. Interconnections between Sigma B, *agr*, and proteolytic activity in *Staphylococcus aureus* biofilm maturation. *Infect Immun* **2009**, *77*, 1623-1635.
62. Kathirvel, M.; Buchad, H.; Nair, M. Enhancement of the pathogenicity of *Staphylococcus aureus* strain Newman by a small noncoding RNA SprX1. *Med Microbiol Immunol* **2016**, *205*, 563-574.
63. Pamp, S.J.; Frees, D.; Engelmann, S.; Hecker, M.; Ingmer, H. Spx is a global effector impacting stress tolerance and biofilm formation in *Staphylococcus aureus*. *J Bacteriol* **2006**, *188*, 4861-4870.
64. Panasenko, O.O.; Bezrukov, F.; Komarynets, O.; Renzoni, A. YjbH solubility controls Spx in *Staphylococcus aureus*: Implication for MazEF Toxin-Antitoxin system regulation. *Front Microbiol* **2020**, *11*, 113.
65. Brown, S.; Santa Maria, J.P.Jr.; Walker, S. Wall teichoic acids of gram-positive bacteria. *Annu Rev Microbiol* **2013**, *67*, 313-336.

66. Holland, L.M.; Conlon, B.; O'Gara, J.P. Mutation of *tagO* reveals an essential role for wall teichoic acids in *Staphylococcus epidermidis* biofilm development. *Microbiology* (Reading) **2011**, *157*, 408-418.
67. Farha, M.A.; Leung, A.; Sewell, E.W.; D'Elia, M.A.; Allison, S.E.; Ejim, L.; Pereira, P.M.; Pinho, M.G.; Wright, G.D.; Brown, E.D. Inhibition of WTA synthesis blocks the cooperative action of PBPs and sensitizes MRSA to β-lactams. *ACS Chem Biol* **2013**, *8*, 226-233.
68. Brown, S.; Xia, G.; Luhachack, L.G.; Campbell, J.; Meredith, T.C.; Chen, C.; Winstel, V.; Gekeler, C.; Irazoqui, J.E.; Peschel, A.; Walker, S. Methicillin resistance in *Staphylococcus aureus* requires glycosylated wall teichoic acids. *Proc Natl Acad Sci U S A* **2012**, *109*, 18909-18914.
69. Ulluwishewa, D.; Wang, L.; Pereira, C.; Flynn, S.; Cain, E.; Stick, S.; Reen, F.J.; Ramsay, J.P.; O'Gara, F. Dissecting the regulation of bile-induced biofilm formation in *Staphylococcus aureus*. *Microbiology* **2016**, *162*, 1398-1406.
70. Donegan, N.P.; Marvin, J.S.; Cheung, A.L. Role of adaptor TrfA and ClpPC in controlling levels of SsrA-tagged proteins and antitoxins in *Staphylococcus aureus*. *J Bacteriol* **2014**, *196*, 4140-4151.
71. Poupel, O.; Proux, C.; Jagla, B.; Msadek, T.; Dubrac, S. SpdC, a novel virulence factor, controls histidine kinase activity in *Staphylococcus aureus*. *PLoS Pathog* **2018**, *14*, e1006917.
